# Supplementary material for: YcgC represents a new protein deacetylase family in prokaryotes
Source: eLife. 2015 Dec 30;4:e05322. doi: 10.7554/eLife.05322 (PMC4709262; doi:10.7554/eLife.05322)
Supplement: Supplementary file 1. — DOI: http://dx.doi.org/10.7554/eLife.05322.016 [file elife-05322-supp1.docx]

**Supplementary File 1.** Genes differentially expressed when YcgC was overexpressed.

| **Gene_ID** | **Gene** | **Ratio (YcgC_OE/WT)** |
| --- | --- | --- |
| GeneID:949083 | lacY | 164.20 |
| GeneID:945006 | lacZ | 95.04 |
| GeneID:945674 | lacA | 24.70 |
| GeneID:945749 | dhaH | 7.59 |
| GeneID:945754 | alr | 5.88 |
| GeneID:947416 | mscS | 4.81 |
| GeneID:946985 | guaB | 4.43 |
| GeneID:945185 | ompT | 4.22 |
| GeneID:948037 | treF | 3.64 |
| GeneID:947165 | proX | 3.22 |
| GeneID:945887 | pspA | 3.20 |
| GeneID:946410 | ftn | 3.10 |
| GeneID:946132 | poxB | 3.01 |
| GeneID:945864 | rnb | 3.00 |
| GeneID:947145 | proW | 2.85 |
| GeneID:948517 | aceA | 2.84 |
| GeneID:1450298 | ytjA | 2.75 |
| GeneID:944905 | queA | 2.63 |
| GeneID:946204 | trpA | 2.61 |
| GeneID:947497 | leuS | 2.59 |
| GeneID:947117 | rfbD | 2.58 |
| GeneID:948800 | ybaQ | 2.56 |
| GeneID:945594 | yccF | 2.53 |
| GeneID:946090 | cspF | 2.51 |
| GeneID:948726 | fklB | 2.51 |
| GeneID:946311 | yeaQ | 2.49 |
| GeneID:944981 | betI | 2.48 |
| GeneID:946822 | yfdI | 2.48 |
| GeneID:944975 | yahK | 2.47 |
| GeneID:945862 | topA | 2.46 |
| GeneID:945857 | yciV | 2.44 |
| GeneID:948044 | ybeZ | 2.43 |
| GeneID:945389 | metK | 2.42 |
| GeneID:948404 | cpxR | 2.42 |
| GeneID:945716 | betA | 2.40 |
| GeneID:945079 | betT | 2.39 |
| GeneID:946031 | sfcA | 2.39 |
| GeneID:946219 | ydiU | 2.38 |
| GeneID:944955 | ltaE | 2.37 |
| GeneID:945865 | tktB | 2.37 |
| GeneID:945786 | ydhS | 2.35 |
| GeneID:945381 | ybhC | 2.35 |
| GeneID:947144 | ygaU | 2.33 |
| GeneID:945459 | yliB | 2.30 |
| GeneID:947376 | betB | 2.30 |
| GeneID:944967 | ompX | 2.28 |
| GeneID:947680 | yhbC | 2.27 |
| GeneID:947548 | rfaE | 2.27 |
| GeneID:945788 | narX | 2.27 |
| GeneID:945067 | thiI | 2.25 |
| GeneID:946614 | gyrA | 2.23 |
| GeneID:946106 | copA | 2.22 |
| GeneID:945276 | rfbB | 2.21 |
| GeneID:2847739 | yfjD | 2.21 |
| GeneID:946794 | purF | 2.20 |
| GeneID:947682 | nusA | 2.20 |
| GeneID:946802 | gdhA | 2.19 |
| GeneID:949048 | kdtA | 2.19 |
| GeneID:945683 | pspF | 2.18 |
| GeneID:947002 | iscU | 2.17 |
| GeneID:945527 | serC | 2.17 |
| GeneID:948906 | yjjU | 2.15 |
| GeneID:945690 | trmU | 2.15 |
| GeneID:948585 | rdgC | 2.14 |
| GeneID:947010 | yfhG | 2.14 |
| GeneID:945564 | ycbY | 2.14 |
| GeneID:948655 | groES | 2.11 |
| GeneID:945766 | fabD | 2.11 |
| GeneID:946468 | dsrB | 2.11 |
| GeneID:946365 | ycgB | 2.10 |
| GeneID:949054 | trxB | 2.10 |
| GeneID:946301 | mipA | 2.09 |
| GeneID:945637 | rluC | 2.08 |
| GeneID:946712 | menF | 2.08 |
| GeneID:945802 | oppD | 2.08 |
| GeneID:946560 | galF | 2.06 |
| GeneID:945457 | yliA | 2.06 |
| GeneID:945870 | fabI | 2.05 |
| GeneID:948216 | rpmH | 2.05 |
| GeneID:945858 | sohB | 2.05 |
| GeneID:945258 | serA | 2.05 |
| GeneID:945154 | rfbA | 2.04 |
| GeneID:945073 | cyoE | 2.04 |
| GeneID:946478 | yedJ | 2.03 |
| GeneID:947138 | yqaE | 2.03 |
| GeneID:945057 | ispF | 2.03 |
| GeneID:945080 | cyoA | 2.03 |
| GeneID:946229 | aroH | 2.01 |
| GeneID:947815 | yhcB | 2.01 |
| GeneID:945217 | lipB | 2.01 |
| GeneID:948512 | aceB | 2.01 |
| GeneID:946906 | gltX | 2.00 |
| GeneID:945226 | purR | 2.00 |
| GeneID:947986 | nikB | 0.50 |
| GeneID:947868 | nirB | 0.50 |
| GeneID:947488 | yqiA | 0.50 |
| GeneID:947355 | yaiZ | 0.50 |
| GeneID:949024 | yehT | 0.49 |
| GeneID:945403 | ybhR | 0.49 |
| GeneID:945829 | hns | 0.49 |
| GeneID:948966 | rmuC | 0.49 |
| GeneID:947155 | nrdE | 0.48 |
| GeneID:947331 | ushA | 0.48 |
| GeneID:945600 | putA | 0.48 |
| GeneID:944856 | fhuA | 0.48 |
| GeneID:948820 \| GeneID:2847727 | mokB\|hokB | 0.48 |
| GeneID:948881 | yjjM | 0.48 |
| GeneID:947671 | yhbQ | 0.48 |
| GeneID:948755 | nrdD | 0.48 |
| GeneID:948636 | melA | 0.48 |
| GeneID:948380 | yihX | 0.47 |
| GeneID:948584 | fdhF | 0.47 |
| GeneID:945545 | ycbJ | 0.47 |
| GeneID:945669 | cspD | 0.46 |
| GeneID:946795 | uxuB | 0.46 |
| GeneID:948902 | deoC | 0.46 |
| GeneID:946338 | yobF | 0.46 |
| GeneID:945435 | glnQ | 0.46 |
| GeneID:948411 | sbp | 0.46 |
| GeneID:947074 | yeaC | 0.46 |
| GeneID:944804 | fruR | 0.46 |
| GeneID:948724 | ytfE | 0.45 |
| GeneID:945252 | ybeL | 0.45 |
| GeneID:948757 | nrdG | 0.45 |
| GeneID:946610 | gatB | 0.45 |
| GeneID:948522 | metH | 0.45 |
| GeneID:946935 | eutQ | 0.45 |
| GeneID:948261 | rbsB | 0.45 |
| GeneID:945214 | fepD | 0.45 |
| GeneID:948367 | yihM | 0.45 |
| GeneID:948823 | yfcY | 0.45 |
| GeneID:945748 | dhaL | 0.44 |
| GeneID:945339 | chbB | 0.44 |
| GeneID:948631 | basR | 0.44 |
| GeneID:945128 | ompW | 0.44 |
| GeneID:944954 | mhpC | 0.44 |
| GeneID:946147 | fumC | 0.44 |
| GeneID:947249 | ygiC | 0.44 |
| GeneID:947313 | lysA | 0.44 |
| GeneID:948737 | ytfJ | 0.44 |
| GeneID:948901 | deoA | 0.44 |
| GeneID:948240 | pstB | 0.44 |
| GeneID:948422 | glpF | 0.44 |
| GeneID:947515 | cpdA | 0.43 |
| GeneID:945672 | aldA | 0.43 |
| GeneID:948137 | htrL | 0.43 |
| GeneID:946334 | manX | 0.43 |
| GeneID:945602 | putP | 0.43 |
| GeneID:945250 | fhuC | 0.43 |
| GeneID:945621 | glnP | 0.43 |
| GeneID:948183 | ilvN | 0.43 |
| GeneID:948068 | eptB | 0.43 |
| GeneID:944756 | mokC | 0.42 |
| GeneID:947192 | hypC | 0.42 |
| GeneID:945290 | nagB | 0.42 |
| GeneID:947262 | sdaB | 0.42 |
| GeneID:946858 | glk | 0.42 |
| GeneID:948505 | zraR | 0.42 |
| GeneID:948680 | frdC | 0.41 |
| GeneID:948324 | fadA | 0.41 |
| GeneID:946546 | sbmC | 0.41 |
| GeneID:945405 | ybhQ | 0.41 |
| GeneID:946865 | yfeD | 0.41 |
| GeneID:944913 | yafM | 0.41 |
| GeneID:946157 | yeaS | 0.41 |
| GeneID:944968 | yahN | 0.41 |
| GeneID:947324 | yqeF | 0.40 |
| GeneID:946898 | ucpA | 0.40 |
| GeneID:945284 | entA | 0.40 |
| GeneID:948603 | rpiR | 0.40 |
| GeneID:947314 | galR | 0.39 |
| GeneID:946345 | exbD | 0.39 |
| GeneID:947018 | hmp | 0.39 |
| GeneID:946332 | manY | 0.39 |
| GeneID:946007 | ydcT | 0.38 |
| GeneID:947264 | sdaC | 0.38 |
| GeneID:949021 | ybdD | 0.38 |
| GeneID:946407 | yecI | 0.38 |
| GeneID:947480 | yghZ | 0.38 |
| GeneID:945431 | dinG | 0.38 |
| GeneID:947189 | hypD | 0.38 |
| GeneID:945292 | nagE | 0.38 |
| GeneID:948104 | aldB | 0.37 |
| GeneID:945358 | galK | 0.37 |
| GeneID:946820 | fadL | 0.37 |
| GeneID:946236 | yniA | 0.36 |
| GeneID:947479 | yqhA | 0.36 |
| GeneID:948729 | cpdB | 0.36 |
| GeneID:946005 | ydcS | 0.36 |
| GeneID:947082 | uxuA | 0.36 |
| GeneID:948423 | glpK | 0.35 |
| GeneID:949011 | atoS | 0.35 |
| GeneID:948520 | pepE | 0.35 |
| GeneID:945130 | pmrD | 0.35 |
| GeneID:947397 | yadI | 0.35 |
| GeneID:945667 | pckA | 0.34 |
| GeneID:947454 | ansB | 0.34 |
| GeneID:946947 | maeB | 0.34 |
| GeneID:945182 | hybC | 0.34 |
| GeneID:945201 | fepC | 0.34 |
| GeneID:948904 | yjjI | 0.34 |
| GeneID:945542 | uxaB | 0.34 |
| GeneID:945227 | lipA | 0.34 |
| GeneID:946242 | tsx | 0.33 |
| GeneID:945213 | cstA | 0.33 |
| GeneID:945789 | yjiM | 0.33 |
| GeneID:948266 | rbsR | 0.32 |
| GeneID:947273 | fucO | 0.32 |
| GeneID:946489 | yeeI | 0.32 |
| GeneID:945192 | yedE | 0.32 |
| GeneID:946592 | hcp | 0.31 |
| GeneID:947881 | nirD | 0.31 |
| GeneID:948761 | treB | 0.31 |
| GeneID:946826 | fumA | 0.31 |
| GeneID:949097 | yfcX | 0.31 |
| GeneID:948668 | frdD | 0.30 |
| GeneID:944830 | gcd | 0.30 |
| GeneID:946316 | yeaT | 0.30 |
| GeneID:946251 | ygjH | 0.29 |
| GeneID:948336 | fadB | 0.29 |
| GeneID:947478 | yghA | 0.28 |
| GeneID:948747 | chpB | 0.28 |
| GeneID:948153 | dinD | 0.28 |
| GeneID:948891 | fhuF | 0.28 |
| GeneID:948237 | pstS | 0.28 |
| GeneID:948878 | hsdR | 0.28 |
| GeneID:948039 | dctA | 0.27 |
| GeneID:945997 | ydcH | 0.27 |
| GeneID:946342 | manZ | 0.27 |
| GeneID:945611 | ndk | 0.26 |
| GeneID:946677 | fruB | 0.26 |
| GeneID:948407 | kdgT | 0.26 |
| GeneID:945747 | dhaK | 0.26 |
| GeneID:948093 | yiaM | 0.26 |
| GeneID:948762 | treC | 0.26 |
| GeneID:945546 | ycbC | 0.26 |
| GeneID:945773 | agp | 0.26 |
| GeneID:948562 | aphA | 0.26 |
| GeneID:945503 | rihA | 0.25 |
| GeneID:946138 | ynfM | 0.25 |
| GeneID:948709 | yjfN | 0.25 |
| GeneID:946718 | yfaH | 0.25 |
| GeneID:945980 | ydbC | 0.25 |
| GeneID:947601 | exuT | 0.25 |
| GeneID:947466 | glcC | 0.25 |
| GeneID:947594 | fadH | 0.25 |
| GeneID:945344 | yfbJ | 0.25 |
| GeneID:948424 | glpX | 0.25 |
| GeneID:948848 | gntP | 0.24 |
| GeneID:948506 | zraS | 0.24 |
| GeneID:946909 | yedF | 0.23 |
| GeneID:944872 | glnH | 0.23 |
| GeneID:947600 | ygjR | 0.22 |
| GeneID:945301 | aer | 0.22 |
| GeneID:948914 | yjiY | 0.22 |
| GeneID:946725 | glpQ | 0.22 |
| GeneID:944796 | rihC | 0.22 |
| GeneID:947104 | malI | 0.21 |
| GeneID:947599 | uxaC | 0.21 |
| GeneID:948221 | tnaA | 0.20 |
| GeneID:948615 | hybB | 0.20 |
| GeneID:948937 | srlD | 0.19 |
| GeneID:945357 | galT | 0.19 |
| GeneID:947922 | malP | 0.19 |
| GeneID:946735 | glpC | 0.19 |
| GeneID:946282 | nupG | 0.18 |
| GeneID:946704 | glpT | 0.17 |
| GeneID:945354 | galE | 0.16 |
| GeneID:948220 | tnaB | 0.16 |
| GeneID:945902 | hybO | 0.16 |
| GeneID:947320 | ygeV | 0.16 |
| GeneID:948971 | srlB | 0.15 |
| GeneID:948572 | acs | 0.14 |
| GeneID:948708 | yjfO | 0.14 |
| GeneID:948933 | srlE | 0.13 |
| GeneID:947487 | fucP | 0.12 |
| GeneID:948223 | tnaC | 0.11 |
| GeneID:945957 | ydeN | 0.10 |
| GeneID:948438 | ykgE | 0.09 |
| GeneID:949041 | mglB | 0.09 |
| GeneID:947575 | srlA | 0.08 |
| GeneID:944842 | hybA | 0.08 |
| GeneID:949039 | mglC | 0.08 |
| GeneID:946319 | yeaU | 0.07 |
| GeneID:948530 | malG | 0.04 |
| GeneID:948547 | malM | 0.03 |
| GeneID:948537 | malK | 0.03 |
| GeneID:948532 | malF | 0.02 |
| GeneID:948538 | malE | 0.01 |
| GeneID:948548 | lamB | 0.00 |
