# Supplementary material for: YcgC represents a new protein deacetylase family in prokaryotes
Source: eLife. 2015 Dec 30;4:e05322. doi: 10.7554/eLife.05322 (PMC4709262; doi:10.7554/eLife.05322)
Supplement: Supplementary file 2. — DOI: http://dx.doi.org/10.7554/eLife.05322.017 [file elife-05322-supp2.docx]

**Supplementary File 2.** Genes differentially expressed when CobB was induced.

| **Gene_ID** | **Gene** | **Ratio (Cob_OE/WT)** |
| --- | --- | --- |
| GeneID:946480 | yodA | 32.57 |
| GeneID:946202 | puuA | 17.43 |
| GeneID:945351 | nadA | 10.90 |
| GeneID:945006 | lacZ | 9.93 |
| GeneID:947591 | ygjN | 8.95 |
| GeneID:945181 | fes | 8.50 |
| GeneID:945687 | cobB | 8.49 |
| GeneID:946006 | yncE | 8.10 |
| GeneID:945882 | puuD | 7.98 |
| GeneID:944960 | ykgM | 7.98 |
| GeneID:945603 | ycdO | 7.89 |
| GeneID:948289 | trxA | 7.49 |
| GeneID:945596 | ycdS | 7.41 |
| GeneID:948279 | ilvM | 6.92 |
| GeneID:1450238 | ykgO | 6.91 |
| GeneID:945214 | fepD | 6.77 |
| GeneID:948891 | fhuF | 6.68 |
| GeneID:947082 | uxuA | 6.52 |
| GeneID:946109 | rem | 6.48 |
| GeneID:947500 | - | 6.36 |
| GeneID:945511 | entC | 6.28 |
| GeneID:945674 | lacA | 6.21 |
| GeneID:945606 | ycdQ | 6.05 |
| GeneID:945983 | ydbD | 5.96 |
| GeneID:948616 | hokD | 5.79 |
| GeneID:949083 | lacY | 5.78 |
| GeneID:946224 \| GeneID:2847683 | tpr\|rtT | 5.61 |
| GeneID:944742 | thrL | 5.59 |
| GeneID:947593 | ygjM | 5.55 |
| GeneID:945492 | ycfR | 5.10 |
| GeneID:948035 | yhjC | 5.10 |
| GeneID:1450289 | yhdL | 5.04 |
| GeneID:949042 | cirA | 4.94 |
| GeneID:945193 | fepA | 4.79 |
| GeneID:946090 | cspF | 4.56 |
| GeneID:945604 | ycdR | 4.51 |
| GeneID:946526 | yeeE | 4.48 |
| GeneID:946345 | exbD | 4.45 |
| GeneID:948554 | dinF | 4.32 |
| GeneID:947549 | relE | 4.27 |
| GeneID:947073 | afuB | 4.24 |
| GeneID:944962 | ykgL | 4.20 |
| GeneID:946702 | mqo | 3.99 |
| GeneID:946375 | znuA | 3.98 |
| GeneID:948429 | hslV | 3.97 |
| GeneID:948283 | ilvL | 3.87 |
| GeneID:949062 | yadS | 3.86 |
| GeneID:945814 | ygiT | 3.84 |
| GeneID:948848 | gntP | 3.83 |
| GeneID:948801 | ydfV | 3.80 |
| GeneID:945371 | mngR | 3.71 |
| GeneID:948747 | chpB | 3.66 |
| GeneID:945843 | tonB | 3.66 |
| GeneID:947459 | yahA | 3.64 |
| GeneID:947161 | nrdH | 3.58 |
| GeneID:948308 | relB | 3.58 |
| GeneID:946932 | yffS | 3.57 |
| GeneID:946048 | yebQ | 3.55 |
| GeneID:948724 | ytfE | 3.50 |
| GeneID:945728 | ymgG | 3.47 |
| GeneID:945847 | yagK | 3.47 |
| GeneID:947215 | iap | 3.37 |
| GeneID:945284 | entA | 3.36 |
| GeneID:947503 | ycdP | 3.32 |
| GeneID:946091 | cspB | 3.32 |
| GeneID:947935 | glpE | 3.30 |
| GeneID:947694 | obgE | 3.27 |
| GeneID:947912 | hslR | 3.23 |
| GeneID:946995 | hscB | 3.22 |
| GeneID:948221 | tnaA | 3.22 |
| GeneID:946770 | cynX | 3.19 |
| GeneID:947252 | chpA | 3.17 |
| GeneID:945194 | entD | 3.06 |
| GeneID:948950 | proQ | 3.05 |
| GeneID:947187 | ygbA | 2.99 |
| GeneID:944858 | yciM | 2.97 |
| GeneID:946111 | ydfW | 2.97 |
| GeneID:945420 | exbB | 2.97 |
| GeneID:948814 | insN-2 | 2.96 |
| GeneID:948336 | fadB | 2.96 |
| GeneID:945066 | nusB | 2.92 |
| GeneID:947760 | aaeR | 2.92 |
| GeneID:947145 | proW | 2.92 |
| GeneID:947148 | proV | 2.91 |
| GeneID:944856 | fhuA | 2.91 |
| GeneID:947178 | hslO | 2.89 |
| GeneID:945029 | ymcD | 2.88 |
| GeneID:946515 | hisI | 2.87 |
| GeneID:946226 | ycgL | 2.86 |
| GeneID:945279 | iscR | 2.86 |
| GeneID:946999 | iscA | 2.85 |
| GeneID:948156 | rph | 2.84 |
| GeneID:948338 | yigI | 2.84 |
| GeneID:947928 | rtcR | 2.82 |
| GeneID:946115 | ycgM | 2.82 |
| GeneID:946551 | hisC | 2.81 |
| GeneID:947699 | yhbE | 2.79 |
| GeneID:946004 | ydcR | 2.77 |
| GeneID:948822 | yfdK | 2.74 |
| GeneID:946425 | proB | 2.72 |
| GeneID:946038 | fdnI | 2.71 |
| GeneID:945150 | allC | 2.70 |
| GeneID:947245 | chpR | 2.70 |
| GeneID:947260 | ybjF | 2.70 |
| GeneID:946359 | umuC | 2.70 |
| GeneID:946415 | yecM | 2.69 |
| GeneID:947411 | yagP | 2.68 |
| GeneID:945436 | yliL | 2.67 |
| GeneID:946350 | yebO | 2.67 |
| GeneID:2847669 | yjiV | 2.65 |
| GeneID:947149 | nrdF | 2.65 |
| GeneID:946374 | znuC | 2.61 |
| GeneID:947025 | yghD | 2.60 |
| GeneID:948443 | clpS | 2.59 |
| GeneID:946924 | eutB | 2.55 |
| GeneID:945308 | yaiP | 2.54 |
| GeneID:944863 | glnD | 2.54 |
| GeneID:946238 | ydjN | 2.53 |
| GeneID:948772 | yjgM | 2.51 |
| GeneID:946412 | tyrP | 2.49 |
| GeneID:948663 | yjeJ | 2.47 |
| GeneID:946467 | rcsA | 2.46 |
| GeneID:944806 | mraW | 2.45 |
| GeneID:945591 | cspG | 2.44 |
| GeneID:947613 | marA | 2.43 |
| GeneID:945732 | ymgD | 2.40 |
| GeneID:947077 | clpB | 2.39 |
| GeneID:949107 | ypdI | 2.39 |
| GeneID:947786 | zntR | 2.38 |
| GeneID:947121 | pyrF | 2.36 |
| GeneID:948685 | yjeF | 2.35 |
| GeneID:945226 | purR | 2.34 |
| GeneID:945007 | lacI | 2.33 |
| GeneID:946014 | yncC | 2.33 |
| GeneID:946340 | rrmA | 2.32 |
| GeneID:946478 | yedJ | 2.32 |
| GeneID:944981 | betI | 2.32 |
| GeneID:949129 | dnaK | 2.31 |
| GeneID:947836 | bfd | 2.29 |
| GeneID:948403 | sodA | 2.28 |
| GeneID:945041 | phoA | 2.27 |
| GeneID:947551 | uppP | 2.26 |
| GeneID:946259 | astB | 2.25 |
| GeneID:948151 | rfaS | 2.25 |
| GeneID:947158 | nrdI | 2.25 |
| GeneID:947785 | yhdN | 2.25 |
| GeneID:2847714 | ryfA | 2.25 |
| GeneID:948381 | yihW | 2.25 |
| GeneID:945250 | fhuC | 2.24 |
| GeneID:947002 | iscU | 2.24 |
| GeneID:945650 | yceD | 2.23 |
| GeneID:944889 | tilS | 2.22 |
| GeneID:947601 | exuT | 2.21 |
| GeneID:946802 | gdhA | 2.21 |
| GeneID:948740 | yzfA | 2.20 |
| GeneID:947925 | gntY | 2.19 |
| GeneID:948044 | ybeZ | 2.19 |
| GeneID:945443 | gltJ | 2.18 |
| GeneID:947968 | ftsE | 2.18 |
| GeneID:944988 | frmA | 2.17 |
| GeneID:945542 | uxaB | 2.17 |
| GeneID:946844 | evgS | 2.16 |
| GeneID:946547 | hisL | 2.16 |
| GeneID:948380 | yihX | 2.15 |
| GeneID:947497 | leuS | 2.15 |
| GeneID:945552 | ycbW | 2.15 |
| GeneID:948147 | rfaK | 2.14 |
| GeneID:948410 | cdh | 2.13 |
| GeneID:948258 | asnA | 2.13 |
| GeneID:948259 | asnC | 2.13 |
| GeneID:945893 | pspB | 2.12 |
| GeneID:1450249 | gnsA | 2.10 |
| GeneID:947417 | ynaK | 2.10 |
| GeneID:948468 | btuB | 2.09 |
| GeneID:947081 | pheA | 2.08 |
| GeneID:947439 | yqgF | 2.08 |
| GeneID:947155 | nrdE | 2.07 |
| GeneID:945273 | erfK | 2.06 |
| GeneID:947888 | yhfY | 2.06 |
| GeneID:945179 | wbbH | 2.06 |
| GeneID:947322 | macA | 2.05 |
| GeneID:946104 | yceP | 2.05 |
| GeneID:948802 | yjgB | 2.04 |
| GeneID:946427 | fecA | 2.04 |
| GeneID:947114 | yohL | 2.04 |
| GeneID:948657 | fxsA | 2.03 |
| GeneID:944756 \| GeneID:2847744 | mokC\|hokC | 2.03 |
| GeneID:945788 | narX | 2.03 |
| GeneID:945001 | cynR | 2.03 |
| GeneID:949089 | yjiR | 2.02 |
| GeneID:947636 | agaR | 2.02 |
| GeneID:945673 | lolC | 2.02 |
| GeneID:945079 | betT | 2.01 |
| GeneID:945693 | ycfC | 2.01 |
| GeneID:948180 | emrD | 2.00 |
| GeneID:946365 | ycgB | 0.50 |
| GeneID:945042 | brnQ | 0.50 |
| GeneID:948757 | nrdG | 0.50 |
| GeneID:946716 | ompC | 0.50 |
| GeneID:949052 | cydD | 0.50 |
| GeneID:947334 | guaA | 0.50 |
| GeneID:948299 | rffH | 0.50 |
| GeneID:947194 | hypB | 0.50 |
| GeneID:948467 | murI | 0.49 |
| GeneID:946675 | yejA | 0.49 |
| GeneID:944813 | murF | 0.49 |
| GeneID:947789 | rsmB | 0.49 |
| GeneID:948266 | rbsR | 0.49 |
| GeneID:948086 | xylR | 0.49 |
| GeneID:945363 | ybaT | 0.49 |
| GeneID:948545 | ubiC | 0.49 |
| GeneID:945319 | yejG | 0.49 |
| GeneID:946023 | yncG | 0.49 |
| GeneID:947527 | ygcF | 0.49 |
| GeneID:947397 | yadI | 0.49 |
| GeneID:948666 | frdB | 0.49 |
| GeneID:947542 | pgpA | 0.49 |
| GeneID:944760 | lysS | 0.48 |
| GeneID:945990 | yncJ | 0.48 |
| GeneID:947731 | nuoM | 0.48 |
| GeneID:948432 | metF | 0.48 |
| GeneID:946826 | fumA | 0.48 |
| GeneID:944866 | tsf | 0.48 |
| GeneID:947263 | wrbA | 0.48 |
| GeneID:945760 | ymgE | 0.48 |
| GeneID:945627 | mdtG | 0.48 |
| GeneID:948242 | atpA | 0.48 |
| GeneID:2847739 | yfjD | 0.47 |
| GeneID:947072 | yffH | 0.47 |
| GeneID:945192 | yedE | 0.47 |
| GeneID:946039 | yafA | 0.47 |
| GeneID:949066 | artM | 0.47 |
| GeneID:948680 | frdC | 0.47 |
| GeneID:944879 | gmhB | 0.47 |
| GeneID:947580 | nuoK | 0.47 |
| GeneID:947129 | yfiA | 0.47 |
| GeneID:945203 \| GeneID:945952 \| GeneID:946118 \| GeneID:946403 \| GeneID:947346 \| GeneID:947517 \| GeneID:948779 \| - | insD-4\|insD-3\|insD-2\|insD-6\|insD-1\|yaiX\|insD-5\|insD-7 | 0.47 |
| GeneID:948245 | atpC | 0.47 |
| GeneID:944745 | yaaJ | 0.47 |
| GeneID:948211 | gyrB | 0.47 |
| GeneID:944814 | mraY | 0.47 |
| GeneID:945545 | ycbJ | 0.47 |
| GeneID:948022 | slp | 0.47 |
| GeneID:948977 | rffD | 0.46 |
| GeneID:945980 | ydbC | 0.46 |
| GeneID:948298 | rffC | 0.46 |
| GeneID:947662 | yraN | 0.46 |
| GeneID:945457 | yliA | 0.46 |
| GeneID:946457 | fliI | 0.46 |
| GeneID:948611 | phnK | 0.46 |
| GeneID:948440 | gldA | 0.46 |
| GeneID:945460 | yliC | 0.46 |
| GeneID:948696 | yjeT | 0.46 |
| GeneID:946762 | nuoG | 0.46 |
| GeneID:947387 | thiL | 0.45 |
| GeneID:948746 | ytfQ | 0.45 |
| GeneID:946600 | yegE | 0.45 |
| GeneID:948323 | metE | 0.45 |
| GeneID:946810 | ydhH | 0.45 |
| GeneID:945435 | glnQ | 0.45 |
| GeneID:945461 | yliD | 0.45 |
| GeneID:946808 | yfcA | 0.44 |
| GeneID:947321 | ymdC | 0.44 |
| GeneID:946209 | ppsA | 0.44 |
| GeneID:946769 | lrhA | 0.44 |
| GeneID:948637 | melR | 0.44 |
| GeneID:946545 | yeeA | 0.44 |
| GeneID:947452 | ybfA | 0.44 |
| GeneID:946761 | nuoH | 0.44 |
| GeneID:945997 | ydcH | 0.44 |
| GeneID:947314 | galR | 0.44 |
| GeneID:944919 | pdxA | 0.44 |
| GeneID:946410 | ftn | 0.43 |
| GeneID:945182 | hybC | 0.43 |
| GeneID:946249 | yfgB | 0.43 |
| GeneID:1450246 | ybgT | 0.43 |
| GeneID:946074 | tam | 0.43 |
| GeneID:948355 | xerC | 0.43 |
| GeneID:944842 | hybA | 0.42 |
| GeneID:1450257 | yciX_2 | 0.42 |
| GeneID:945540 | nuoL | 0.42 |
| GeneID:948080 | glyS | 0.42 |
| GeneID:948488 | rpoB | 0.42 |
| GeneID:948605 | phnI | 0.42 |
| GeneID:946319 | yeaU | 0.42 |
| GeneID:947454 | ansB | 0.41 |
| GeneID:945600 | putA | 0.41 |
| GeneID:947356 | ygfJ | 0.41 |
| GeneID:945459 | yliB | 0.41 |
| GeneID:948064 | dppC | 0.40 |
| GeneID:947788 | trkA | 0.40 |
| GeneID:948686 | yjeS | 0.40 |
| GeneID:945025 \| GeneID:945967 \| GeneID:948781 \| GeneID:946455 \| GeneID:947347 \| GeneID:947520 \| - | insC-4\|insC-3\|insC-2\|insC-6\|insC-1\|yaiX\|insC-5 | 0.40 |
| GeneID:946812 | mepA | 0.40 |
| GeneID:945128 | ompW | 0.39 |
| GeneID:945136 | nuoN | 0.39 |
| GeneID:948517 | aceA | 0.39 |
| GeneID:948648 | yjdI | 0.39 |
| GeneID:947024 | cspE | 0.38 |
| GeneID:948018 | arsC | 0.38 |
| GeneID:948039 | dctA | 0.38 |
| GeneID:948710 | aidB | 0.38 |
| GeneID:945783 | narK | 0.37 |
| GeneID:945268 | ynfF | 0.35 |
| GeneID:945358 | galK | 0.35 |
| GeneID:947434 | galP | 0.34 |
| GeneID:946759 | nuoC | 0.34 |
| GeneID:945519 | trpC | 0.34 |
| GeneID:945789 | yjiM | 0.34 |
| GeneID:946935 | eutQ | 0.33 |
| GeneID:947230 | cysH | 0.32 |
| GeneID:946322 | ftsW | 0.32 |
| GeneID:948085 | yiaI | 0.32 |
| GeneID:2847725 | ytfR | 0.31 |
| GeneID:949037 | yeiA | 0.31 |
| GeneID:948749 | ydhY | 0.31 |
| GeneID:946909 | yedF | 0.31 |
| GeneID:948615 | hybB | 0.30 |
| GeneID:948756 | ydhV | 0.30 |
| GeneID:946175 | lpp | 0.29 |
| GeneID:948617 | phnF | 0.27 |
| GeneID:946732 | nrdB | 0.27 |
| GeneID:948584 | fdhF | 0.25 |
| GeneID:946204 | trpA | 0.19 |
| GeneID:945057 | ispF | 0.17 |
| GeneID:945357 | galT | 0.09 |
| GeneID:949041 | mglB | 0.09 |
| GeneID:945354 | galE | 0.09 |
| GeneID:949043 | galS | 0.07 |
| GeneID:949036 | mglA | 0.04 |
| GeneID:949039 | mglC | 0.03 |
