# Supplementary material for: YcgC represents a new protein deacetylase family in prokaryotes
Source: eLife. 2015 Dec 30;4:e05322. doi: 10.7554/eLife.05322 (PMC4709262; doi:10.7554/eLife.05322)
Supplement: Supplementary file 3. — DOI: http://dx.doi.org/10.7554/eLife.05322.018 [file elife-05322-supp3.docx]

**Supplementary File 3**. Clones constructed in this study.

| **Strain** | **Characterization** |
| --- | --- |
| *E. coli W3110* | W3110 Δ*YcgC*::Km |
| *E. coli BL21* | DhaM of *Shigella flexneri*, pET-28a+ |
| *E. coli BL21* | DhaM of *Klebsiella oxytoca*, pET-28a+ |
| *E. coli BL21* | DhaM of *Pantoea ananatis*, pET-28a+ |
| *E. coli BL21* | DhaM of *Enterocolitica WA-314*, pET-28a+ |
| *E. coli BL21* | DhaM of *Providencia stuartii MRSN*, pET-28a+ |
| *E. coli BL21* | RutR 52 K to Q, pET-28a+ |
| *E. coli BL21* | RutR 62 K to Q, pET-28a+ |
| *E. coli BL21* | RutR 52,62 K to Q, pET-28a+ |
| *E. coli BL21* | RutR 52 K to R, pET-28a+ |
| *E. coli BL21* | RutR 62 K to R, pET-28a+ |
| *E. coli BL21* | RutR 52,62 K to R, pET-28a+ |
| *E. coli BL21* | YcgC 8,10,73,77,200 S to A, pET-28a+ |
| *E. coli BL21* | YcgC 200 S to A, pET-28a+ |
