# Supplementary material for: YcgC represents a new protein deacetylase family in prokaryotes
Source: eLife. 2015 Dec 30;4:e05322. doi: 10.7554/eLife.05322 (PMC4709262; doi:10.7554/eLife.05322)
Supplement: Supplementary file 4. — DOI: http://dx.doi.org/10.7554/eLife.05322.019 [file elife-05322-supp4.docx]

**Supplementary File 4**. Primers used in this study.

| **ID** | **Gene** | **Forward Primer (5'-3')** | **Reverse Primer (5'-3')** |
| --- | --- | --- | --- |
| 1 | ycgC | GCGAAAGGGCCAGAAGCT | GAACGGGACGCAGAGTAGGTG |
| 2 | rutR | GCGATCAAAGAGTACATCCGTC | CGCCTGCCAGCATCTCCATA |
| 3 | gcd | AGCGCATCTGGTGGAACATC | AGATTTGAGCGGTGCGG |
| 4 | pmrD | CCAGCATCGCAGAGCATAAG | GCCATTCCATTGCCGTTG |
